# LDL mediated delivery of Paclitaxel and MRI imaging probes for personalized medicine applications

**DOI:** 10.1186/s12951-021-00955-9

**Published:** 2021-07-13

**Authors:** Sahar Rakhshan, Diego Alberti, Rachele Stefania, Valeria Bitonto, Simonetta Geninatti Crich

**Affiliations:** Department of Molecular Biotechnology and Health Sciences, University of Torino, via Nizza 52, Torino, Italy

**Keywords:** Low density lipoproteins (LDL), Magnetic resonance imaging (MRI), Theranostic, Paclitaxel, Gd based contrast agents

## Abstract

**Background:**

The combination of imaging and therapeutic agents in the same smart nanoparticle is a promising option to perform a minimally invasive imaging guided therapy. In this study, Low density lipoproteins (LDL), one of the most attractive biodegradable and biocompatible nanoparticles, were used for the simultaneous delivery of Paclitaxel (PTX), a hydrophobic antitumour drug and an amphiphilic contrast agent, Gd-AAZTA-C17, in B16-F10 melanoma cell line. These cells overexpress LDL receptors, as assessed by flow cytometry analysis.

**Results:**

PTX and Gd-AAZTA-C17 loaded LDLs (LDL-PTX-Gd) have been prepared, characterized and their stability was assessed under 72 h incubation at 37 °C and compared to LDL loaded with Gd-AAZTA-C17 (LDL-Gd) and LDL-PTX. The cytotoxic effect of LDL-PTX-Gd was evaluated by MTT assay. The anti-tumour drug loaded into LDLs showed a significantly higher toxicity on B16-F10 cells with respect to the commercially available formulation Paclitaxel kabi (PTX Kabi) used in clinical applications. Tumour cells uptake was initially assessed by ICP-MS and MRI on B16-F10 cell line. By the analysis of the image signal intensity, it was possible to extrapolate the amount of internalized PTX indirectly by the decrease of relaxation times caused by Gd, proportional to its concentration. Finally, the treatment with PTX loaded LDL on B16-F10 tumour bearing mice resulted in a marked reduction of tumour growth compared to the administration of PTX Kabi alone.

**Conclusions:**

LDLs are selectively taken-up by tumour cells and can be successfully exploited for the selective delivery of Paclitaxel and imaging agents. For the first time the anon invasive “in vivo” determination of the amount of PTX accumulated in the tumour was possible, thanks to the use of theranostic agents of natural origin.

**Graphic abstract:**

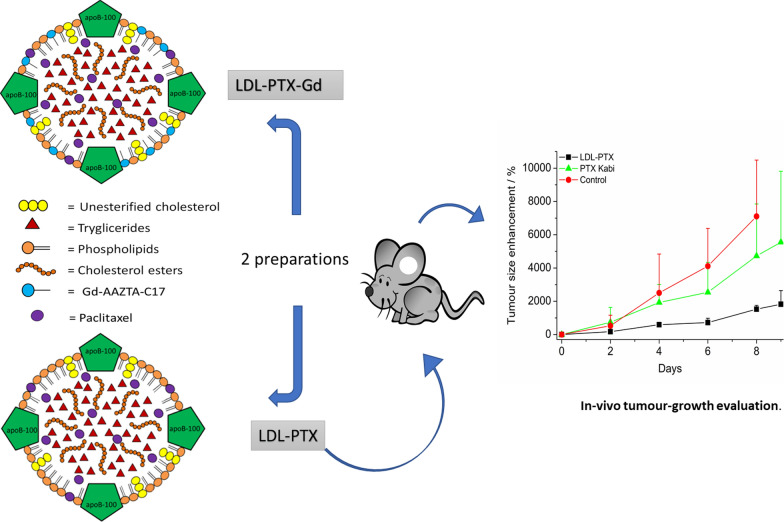

**Supplementary Information:**

The online version contains supplementary material available at 10.1186/s12951-021-00955-9.

## Introduction

**Fig. 1:**
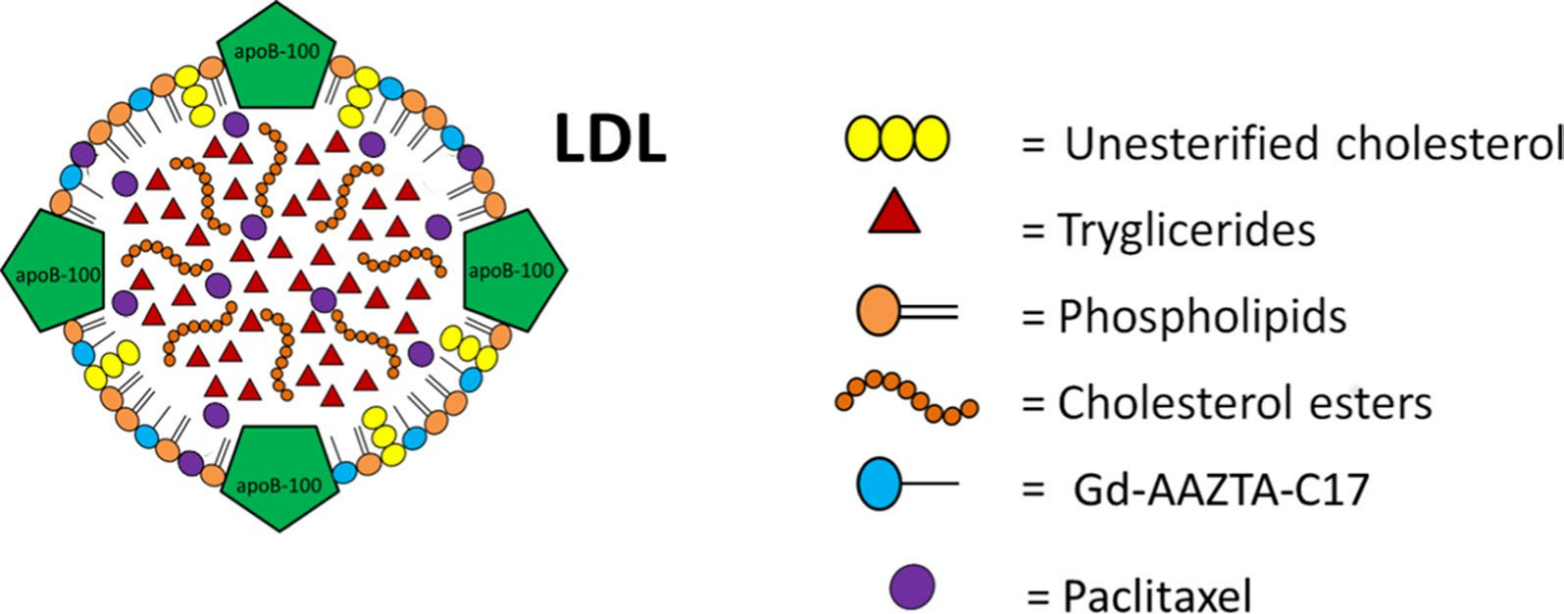
Schematic representation of LDL as a carrier for both PTX and Gd-AAZTA-C17

Nanomedicine [1, 2] is the branch of medicine based on the use of nano-sized carrier materials with the aim to improve the drug biodistribution. Nanomedicine aims to reduce side effects as a consequence of the more specific accumulation at the pathological site and lower concentration in healthy tissues [3]. The nanoparticle commonly used in nanomedicine are liposomes, polymers (i.e. polylactic and glycolic polymers), micelles, and antibodies [4]. In most cases, nanocarriers have also the advantage of increase the solubility, stability and bioavailability of hydrophobic compounds in physiological conditions [5]. Moreover, nanoparticles can deliver imaging probes to perform imaging guided drug delivery [6, 7]. Paclitaxel (PTX) has demonstrated an effective cytotoxic activity against many tumours such as ovarian cancer, breast cancer, mesothelioma, non- small cell lung cancer and melanoma [8]. The main drawback is its extremely low solubility in water (< 0.5 mg/l). The commercially available formulation (Taxol) of PTX has many side effects and results neurotoxic and nephrotoxic [9]. In this contest much effort has been devoted to the development of a biodegradable carriers of natural origin capable of providing a lower and localized dose of drug to the tumour site [10]. In our previous papers, we proposed Low Density Lipoproteins (LDL) as nanoparticles of natural origin for drug and imaging agents delivery [11–13]. LDL are endogenous nanoparticles devoted to the transport of triglycerides, cholesterol, cholesterol esters within the body. Despite the use of proteins as a drug delivery platform, can be advantageous because of their good tolerability, biodegradability and low immunogenity [14–16], endogenous nanosystems have not yet received great attention in clinical practice. The main advantage of their use is the possibility of the exploitation of their natural target receptors that are up-regulated in aggressive tumours such us melanoma, glioma, breast cancer [17–20]. In this study, we propose the use of LDL loaded with Paclitaxel and a lipophilic MRI contrast agent (Gd-AAZTA-C17) to target selectively B16-F10 melanoma cells (Fig. 1). These tumour cells overexpress LDL receptors (LDLR) as demonstrated by a cytofluorimetric assay. The synthesis and characterization of the LDL adduct was described together with cytotoxicity tests (MTT). Finally, the biocompatibility and treatment of solid melanoma tumours obtained by the subcutaneous injection of B16 was assessed and compared with the effect of Paclitaxel kabi (PTX-Kabi, Fresenius Kabi), the water suspended drug formulation used in clinical studies.

## Materials and methods

**Fig. 2:**
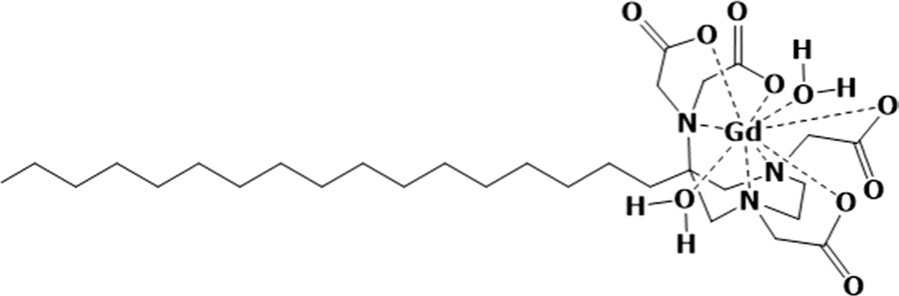
Schematic representation of the Gd based contrast agent Gd-AAZTA- C17

Gd-AAZTA-C17 [21] (AAZTA-C17 = 6-bis(carboxymethyl)amino-4-carboxymethyl-6-heptadecyl-1,4-diazepan-1-yl]acetic acid, Fig. 2) was purchased by Cage Chemicals Srl (Novara, Italy).

### Preparation of LDL-Gd, LDL-PTX and LDL-PTX-Gd adducts

LDL loaded with PTX (LDL-PTX) nanoparticles were prepared by adding dropwise 40 μl of PTX dissolved in DMSO (53 mM) to a stirred suspension of LDL in PBS (2.15 ml). After 4 h incubation at 37 °C, the non-encapsulated drug was removed by dialysis (MW cut off 14 KDa) against PBS (Phosphate-Buffered Saline, pH 7.4, 1.5 L) at 4 °C for 16 h. The LDL loaded with Gd-AAZTA-C17 complexes (LDL-Gd) was prepared as described in a previous paper [22]. Briefly, LDL loaded with Gd-AAZTA-C17 complexes (LDL-Gd) and LDL loaded with both Gd-AAZTA-C17 and PTX (LDL-PTX-Gd) were prepared by adding dropwise 3.8 ml of Gd-AAZTAC17 dissolved in PBS (0.09 mM, below critical micellar concentration) to a stirred suspension of LDL or LDL-PTX (0.6 μM, 2 ml) at 37 °C for 2 h. The unbound complex was eliminated by dialysis as described before. Finally, the LDL adducts were concentrated using Vivaspin centrifuge filters (molecular weight cut-off 50,000, Sartorius) to be used for in vitro and in vivo studies.

### Protein concentration determination

The amount of LDL protein was measured using the colorimetric commercially available Bradford Protein Assay (Biorad). Firstly, a calibration curve was made using a Bovine Serum Albumin (BSA) standard solution. The absorbance was measured at 595 nm using a JenWay 6715 UV/VIS spectrophotometer (Essex, UK).

### Relaxometric measurements

The water proton longitudinal relaxation rates (R1 = 1/T_1_) of LDL-Gd and LDL-PTX-Gd solutions were measured with a Spinmaster relaxometer (Stelar S.r.l., Mede, Italy) at 21.5 MHz and 25 °C. To measure the amount of Gd loaded in the LDLs, a glass vial containing 100 μl of HCl 37% and 100 μl of sample solution was placed in an oven at 110 °C for 16 h. Upon this treatment, all Gd is dissolved as the free aquo-ion. The Gd^3+^ concentration was determined using a calibration curve obtained using standard GdCl_3_ solutions (0.01–2 mM). The method was double-checked by ICP-MS element-2 (Thermo-Finnigan, Rodano, Italy).

### HPLC determination of Paclitaxel content in nanoparticles

In order to determine the encapsulation efficiency of Paclitaxel in LDL, 100µL aliquot of LDL-PTX or LDL-PTX-Gd was mixed with 300 µL of acetonitrile. The sample was then filtered by Phree Phospholipid Removal 1 ml tube (Phenomenex, Torrance, CA, USA) and analysed by Waters Alliance 2695 HPLC system with Waters 2998 Photodiode Array (PDA) Detector, using a SunFire C18 Column, 100 Å, 3.5 µm, 4.6 mm × 150 mm and 0.1% TFA in water (solvent A) and 0.1% TFA in acetonitrile (solvent B). Elution was carried out with a linear 40% to 100% gradient of solvent B into A over 23 min at a 1 ml /min flow rate and a retention time of 10.8 min. The detection wavelength was set at 227 nm. Sample solution was injected at a volume of 25 μl. The HPLC was calibrated with standard solutions of 0.5 to 17 μg/ml of PTX dissolved in acetonitrile (correlation coefficient of R^2^ = 0.9992). The encapsulation efficiency was defined by the ratio of measured and initial amount of PTX encapsulated in nanoparticles.

### Determination of nanoparticles size

The LDL-Gd, LDL-PTX-Gd and LDL-PTX adducts hydrodynamic mean diameter was determined using a Malvern dynamic light-scattering spectrophotometer (Malvern Instruments, Malvern, UK). All samples were analyzed at 25^◦^C in filtered (cut-off = 0.2 μm) PBS buffer (pH 7.4).

### In vitro PTX and Gd-AAZTA-C17 release

To perform stability tests, LDL-PTX and LDL-PTX-Gd nanoparticles were dispersed in 2 ml of PBS at a 0.6 µM LDL and placed in a dialysis bag (MWCO = 14 kD). The samples underwent dialysis against 1 L of PBS at 37 °C with slight agitation via mechanical stirring. At various time intervals (24, 48, 72 h), 200 μl of sample was collected for measurement of size, protein, PTX and Gd concentration, as described before, by RP-HPLC and relaxometry, respectively. The stability of nanoparticles for PTX and Gd concentration were calculated as percentage difference measured with respect to t = 0 set at 100%.

### Cell lines

Mouse melanoma (B16-F10) cell line was obtained from the American Type Culture Corporation (ATCC) and they were cultured in DMEM (Lonza) supplemented with 4.5 g/l glucose, 4 mM glutamine and 10% FBS (Lonza). The medium contained 100 U/ml penicillin and 100 U/ml streptomycin. Cell line was maintained in a humidified incubator at 37 °C, 5% CO_2_.

### FACS analysis of LDL receptor expression

In order to perform FACS (Fluorescence Activated Cell Sorting) analysis, 3 × 10^5^ B16-F10 cells were seeded in 6 cm dishes. After 24 h incubation in 37 °C, the cells were incubated for 24 h with their culture media supplemented with 10% LPDS (Lipoprotein Deficient Serum, Biomedical Technologies Inc., Stoughton, MA). Finally, the cells were washed with PBS, detached with trypsin/EDTA, transferred in 15 ml falcon tubes and counted in PBS. Cell number was determined using a cell sorting chamber (Burker-Turk chamber). B16-10 cells were divided in 3 falcon tubes containing 1 × 10^6^ cells. [Sample 1: no antibody incubated (control); sample 2: secondary antibody incubated; sample 3: primary and secondary antibodies incubated]. All cells were fixed with 4% paraformaldehyde (7 min, 1 ml); then 2 ml of PBS were added to the cells before centrifugation (1100 rpm for 5 min). After a further washing with 3 ml of PBS, cells were permeabilized with 0.1% (v/v) of Tween20 in PBS (20 min, 1 ml); then 2 ml of PBS were added to the cells and they were centrifuged (1100 rpm for 5 min); the PBS was removed, and the cells were further washed with 3 ml of PBS and centrifuged (1100 rpm for 5 min). All the cells were then incubated at 4 °C in 10% FBS (v/v) in PBS (10 min, 1 ml) to block nonspecific protein protein interaction. Then 2 ml of PBS were added to the cells and they were centrifuged (1100 rpm for 5 min); after a further washing with 3 ml of PBS the primary antiLDL receptor monoclonal antibody (Abcam ab52818) was incubated 30 min at 4 °C to cell samples n° 3 (3 µL in 200 μl of 0.1% BSA/PBS). Then 3 ml of PBS were added to the cells and they were centrifuged (1100 rpm for 5 min); the PBS was removed, and the cells were further washed with 3 ml of PBS and centrifuged (1100 rpm for 5 min). Then, a swine antirabbit secondary antibody FITC-conjugated (Dako F0205) (3 μl in 200 μl of 0.1% BSA/PBS) was incubated to samples 2 and 3. The incubation was performed at 4 °C for 30’. All samples were washed with PBS (3 ml) and centrifuged (1100 rpm for 5 min). Finally, all the cells were diluted in PBS (250 µL) and evaluated for their FITC fluorescence on a flow cytometer (Becton Dickinson, FACS Calibur).

### MTT assay

2.5 × 10^3^ B16-F10 cells/well were incubated in a 96-well microtiter plate at 37 °C and 5% CO_2_ atmosphere in their normal medium and after 24 h incubation the medium was changed with medium containing 10% LPDS. 24 h later, the medium was replaced with a fresh medium containing LDL-PTX adducts, PTX-kabi (Fresenius-Kabi) or LDL-PTX-Gd adducts (0.1–10 µM PTX) and incubated for 24 h at 37 °C and 5% CO_2_. After this time, the incubation medium was eliminated and 0.45 mg/ml of MTT labeling reagent (Sigma) dissolved in medium was added into each well and the plates were incubated for 4 h at 37 °C and 5% CO_2_ [19]. Then, after removing the medium containing MTT reagent, 150 µL of the solubilization solution (DMSO) were added into each well to solubilize the formazan salt crystals produced by the metabolism of live cells and the microplate was incubated 30 min at room temperature. Finally, absorbance was read at 570 nm with IMark microplate reader (Biorad). Cell vitality was reported as percentage of cell vitality observed in treated samples relative to that observed in control cells. The experiment was performed in triplicate and the data were graphically presented as mean ± SD.

### Uptake experiments

For the in vitro uptake experiments, 2 × 10^5^ B16-F10 cells were seeded in 6 cm diameter dishes. The day after, the cells were incubated for 24 h with the respective culture media supplemented with 10% LPDS to increase LDLR expression. Finally, the cells were incubated for 24 h with Gd loaded LDL particles (LDL-Gd) at 20 and 50 µg/ml LDL concentration and PTX-Gd-AAZTA loaded LDL particles at 10, 20 and 30 µg/ml LDL. At the end of the incubation, cells were washed three times with 10 ml ice-cold PBS and detached with trypsin/EDTA. Cells were also transferred into glass capillaries for MRI analysis (see below). After MRI analysis, the samples were resuspended in 0.2 ml PBS and sonicated in ice for 30’’ at 30% power. The amount of proteins, proportional to the number of cells of each cell sample lysate, was evaluated by the Bradford assay (see above). Gd content of B16-F10 was determined using inductively coupled plasma mass spectrometry (ICP-MS). Sample digestion was performed with 0.2 ml of concentrated HNO_3_ (70%) added in 0.2 ml of sample lysate using a high performance Microwave Digestion System (ETHOS UP Milestone, Bergamo, Italy). After digestion, the volume of each sample was brought to 3 ml with ultrapure water and the sample was analyzed by ICP-MS.

### Evaluation of LDL-PTX-Gd particles uptake in vitro and in vivo by magnetic resonance imaging (MRI)

MR images were acquired on a Bruker Avance Neo 300 MHz spectrometer (7 T) equipped with a Micro 2.5 microimaging probe (Bruker BioSpin, Ettlingen, Germany). In vitro glass capillaries containing B16-F10 cells were placed in an agar phantom and MR imaging was performed using a standard T_1_-weighted multi-slice spin- echo sequence (TR/TE/NEX = 250/5.04/6, FOV = 1.2 cm). T_1_ relaxation times were calculated using a standard Saturation Recovery Spin Echo.

In vivo adult male C57BL/6J mice were maintained under specific pathogen-free conditions at the animal facility of the Department of Molecular Biotechnology and Health Sciences (University of Turin, Italy). All animal experiments have been carried out in accordance with the EU Directive 2010/63/EU for animal experiments. The animal treatment protocol was approved by the Italian Ministry of Health (Authorization Number 1012/2015-PR). B16-F10 cells were cultured as described above and tumours were generated by the injection of 0.5 × 10^6^ B16-F10 cells in a final volume of 0.15 ml PBS subcutaneously in the neck of the mouse. Seven days after the B16-F10-cell injection mice developed solid tumours of approximately 42 ± 26 mm^3^ in volume, Tumour bearing mice (n = 3) received (iv) a bolus of LDL-PTX-Gd at a dose of 35 μmol/kg Gd. T_1_-weighted spin-echo MR-images (TR/TE/NEX = 200:4:8, FOV = 3 cm) were acquired in pre-anesthetized mice by injecting tiletamine/zolazepam (Zoletil 100; Virbac, Milan, Italy) 20 mg/kg + xylazine (Rompun; Bayer, Milan, Italy) 5 mg/kg before and 6, and 24 h after the particles injection. The mean signal intensity (SI) enhancement (%) in the regions of interest (ROI) manually drawn on the whole tumour, and on the muscle, kidneys, spleen and liver, at the different time intervals was calculated accordingly to the following equation (Eq. 1) and the mean measured SI was normalized using a tube containing standard Gd solution:

1 $${\text{SI}}\% \;{\text{Enhancement}} = \left( {\left( {{\text{mean SI}}_{{{\text{POST}}}} - {\text{mean SI}}_{{{\text{PRE}}}} } \right)/{\text{mean SI}}_{{{\text{PRE}}}} } \right) \times {1}00$$

The concentration of the Gd complex in the tumour and different tissues and organs was calculated from Eq. 2:

2 $$\frac{{SI_{PRE}^{{}} }}{{SI_{POST} }} = \frac{{ \left\{ {\left[ {1 - exp\left( { - TR - TE} \right)R1_{PRE} } \right]} \right\}\exp \left( { - TExR2} \right)}}{{\left\{ {\left[ {1 - exp\left( { - TR - TE} \right)R1_{POST} } \right]} \right\}\exp \left( { - TExR2} \right)}}$$

In Eq. 2 [23], TR is the repetition time, TE is the echo time, and R_1_ and R_2_ are the water proton relaxation rates.

The R_1_ precontrast (R1(pre)) maps were obtained by using a SNAP sequence; R1 postcontrast (R1(post)) maps were calculated by using the pre- and postcontrast SI ratio (Eq. 2) calculated in the regions of interest, which were manually drawn on T_**1**_-weighted images; r1p (in cell) is the intracellular relaxivity of LDL-PTX-Gd (6.14 mM^−1^ s^−1^ at 7 T) and it was used to calculate the intra-tumour Gd concentration.

### In vivo tumour growth assessment and histological studies upon LDL-PTX or PTX kabi treatment in administered mice

When mice developed solid tumours of 42 ± 26 mm^3^, they received an intravenous injection of LDL-PTX (n = 5) or PTX Kabi (n = 7) (corresponding to a concentration of 4 mg/kg PTX). Treated mice were compared to untreated control mice (n = 8). Mice were treated for five days consecutively once a day and the tumour growth were monitored with MRI at 1 T on an Aspect M2-High Performance MRI System (Aspect Magnet Technologies Ltd., Netanya, Israel) every 2 days. The T_2_-weighted MR images were acquired at 1 T with an Aspect M2-High Performance MRI System (Aspect Magnet Technologies Ltd., Netanya, Israel) consisting of a NdFeB magnet, equipped with a 35 mm solenoid Tx/Tr coil of inner diameter 35 mm. This system is equipped with fast gradient coils (gradient strength, 450 mT m − 1 at 60 A: ramp time, 250 μs at 160 V) with a field homogeneity of 0.2–0.5 gauss. The MR images were performed by using a T_2_-weighted protocol (TR/TE/NEX = 2500:50:6; FOV = 4.0 cm). Animals were anesthetized before MRI examination by injecting tiletamine/zolazepam (20 mg/kg; Zoletil 100, Virbac, Milan, Italy) and xylazine (5 mg/kg; Rompun, Bayer, Milan, Italy). The tumour volume (mm^3^) was calculated in the region of interest (ROI) manually drawn on the whole tumour by ITK-SNAP software. Tumour volume enhancement (%) was calculated according to the Eq. 3

3 $$(\left( {{\text{tumour volume}}\left( {{\text{time}} = {\text{n}}} \right){-}{\text{tumour volume}}\left( {{\text{time}} = 0} \right)/{\text{tumour volume}}\left( {{\text{time}} = 0} \right)} \right) \times {1}00$$

where time = 0 indicates the day of the first treatment and time = n indicates the days when mice were acquired by MRI. At the end of the experiment, mice were sacrificed and untreated tumour (control) or tumour treated with LDL-PTX or PTX-Kabi were explanted, fixed in 4% paraformaldehyde for 16 h and then were embedded in paraffin. Dewaxed 5 um sections were stained with hematoxylin–eosin. Images of tumour tissue were acquired using an optical microscope (Olympus BX41).

## Results

### Preparation of LDL-Gd, LDL-PTX and LDL-PTX-Gd

The preparation of LDL-PTX and LDL-PTX-Gd nanoparticles was performed using the procedure described in materials and methods. The recovery of LDL (recovery = % of the protein remaining in the solution after the loading process), the PTX encapsulation efficiency and the adduct sizes, measured by dynamic light scattering (DLS) are reported in Table 1. The size of all the LDL adducts were similar to native LDL (23 ± 1 nm) thus indicating that the loading process does not change dramatically the protein tridimensional structure. The LDL recovery when PTX and Gd-AAZTA-C17 were encapsulated alone was > 44%. When LDL-PTX adducts were loaded with Gd-AAZTA-C17, both the LDL recovery and the encapsulation efficiencies decreased. In this formulation, the quantification of PTX by RP-HPLC measurements showed lower drug incorporation corresponding to a number of Paclitaxel molecules per LDL of 97 ± 17, maybe due to replacements effects. The size of LDL-PTX-Gd adduct measured by DLS was 34 ± 6 nm (Additional file 1: Figure S1), The millimolar relaxivity of LDL-PTX-Gd adduct (r_1p_; the observed relaxation rate of a water solution containing 1 mM of a paramagnetic species) was 25 ± 4.4 mM^*−*1^ s^*−*1^ (21.5 MHz, 25 ^*◦*^C), 30% lower than the r_1p_ found for LDL-Gd.

**Table 1:** Characterization of LDL-PTX, LDL-Gd and LDL-PTX-Gd nanoparticles

NPs	LDL% recovery ± SD	PTX% recovery ± SD	Gd% recovery ± SD	LDL/PTX Molar ratio ± SD	Size nm ± SD	LDL/Gd Molar ratio ± SD	Relaxivity mM^−1^ s^−1^ ± SD
LDL-PTX	44 ± 5	12 ± 2	–	1:262 ± 29	26.5 ± 4		
LDL-Gd	62 ± 11	–	63 ± 5	–	22.8 ± 2.5	1:284 ± 24	31 ± 4.7
LDL-PTX-Gd	24 ± 11	4 ± 1	22	1:97 ± 17	34 ± 6	1:186 ± 7	25 ± 4.4

### Stability of LDL-PTX and LDL-PTX-Gd adducts

**Fig. 3:**
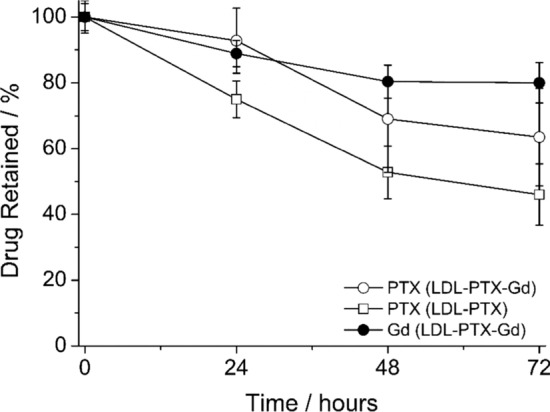
In vitro release of PTX loaded in LDL-PTX-Gd and LDL-PTX, in addition to the release of Gd-AAZTA-C17 loaded in LDL-PTX-Gd, measured at different times (24, 48, 72 h) under dialysis in PBS at 37 °C, pH = 7.4. Error bars correspond to the ± SD obtained by 4 independent experiments

The in vitro release profile of Gd in LDL-PTX-Gd and PTX in LDL-PTX and LDL-PTX-Gd was investigated by RP-HPLC measurements of samples dialyzed at 37 °C in PBS for 24, 48, 72 h. The cumulative percentage release is shown in Fig. 3. PTX release in the LDL-PTX was higher (almost 55%, in 72 h) than in LDL-PTX-Gd (35%, 72 h). Gd-AAZTA-C17 showed higher stability in the LDL adduct (20% release in 72 h). This high stability of PTX in LDL-PTX-Gd (> 90% after 24 h at 37 °C) allows to perform uptake studies in B16-F10 cells. We can surmise that the lower PTX release observed in the LDL adduct containing both PTX and Gd-AAZTA-C17 is due to the exchange of the more unstable PTX molecules located on the external protein surface by Gd-AAZTA-C17 due to its higher affinity for the same binding sites present on the protein surface. In fact, due to its high hydrophobicity, it was hypothesized that PTX is in part located in the internal core and in part in the external surface of the protein.

### Evaluation of low-density lipoproteins receptors (LDLRs) expression on melanoma cell lines

**Fig. 4:**
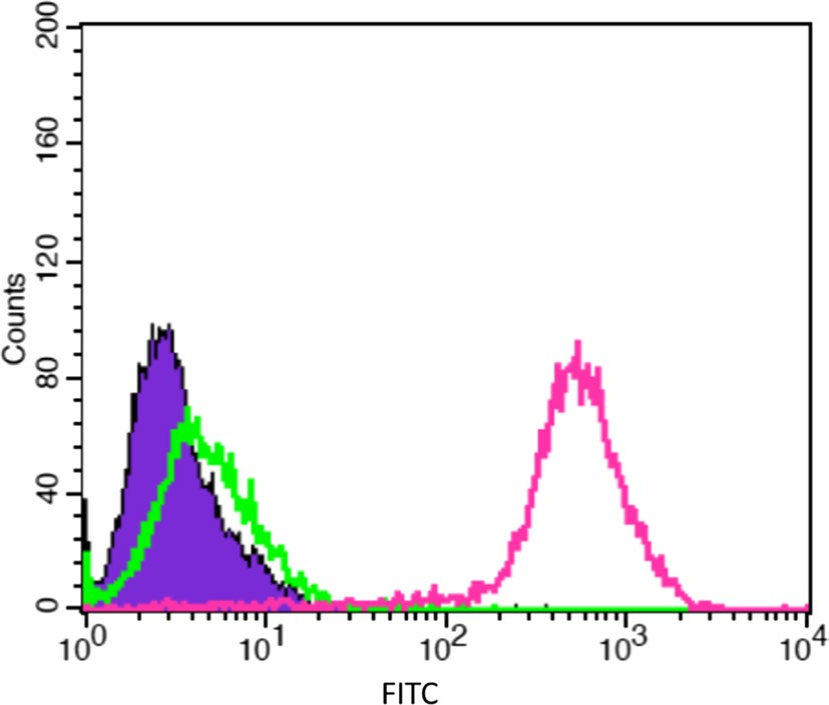
FACS analysis of the LDL receptors expression on B16-F10 cell lines. Representative panel shows the fluorescence intensity of LDL receptors expression on B16-F10 cell line. The positivity is defined as fluorescence intensity of FITC (antiLDL receptor Ab conjugated with FITC secondary Ab) (pink line) higher than that of the FITC secondary Ab used as control (green line). Untreated cells are shown in violet

The LDLRs expression by B16-F10 murine melanoma cell line was evaluated by Flow cytometry (FACS) analysis. Cells were incubated 30’ with a monoclonal antibody (mAb) specific for LDLRs and then with the FITC-labeled secondary Ab (FITC = fluorescein isothiocyanate) (Fig. 4). Untreated cell samples (violet area) were used as control. The specific binding of the secondary Ab was determined incubating cells avoiding pre-incubation with the primary Ab (green line). AntiLDL receptors mAb yielded a high positive signal in B16-F10 cells thus demonstrating a high level of expression of LDLRs (pink line).

### Toxicity Test of PTX-LDL adducts

**Fig. 5:**
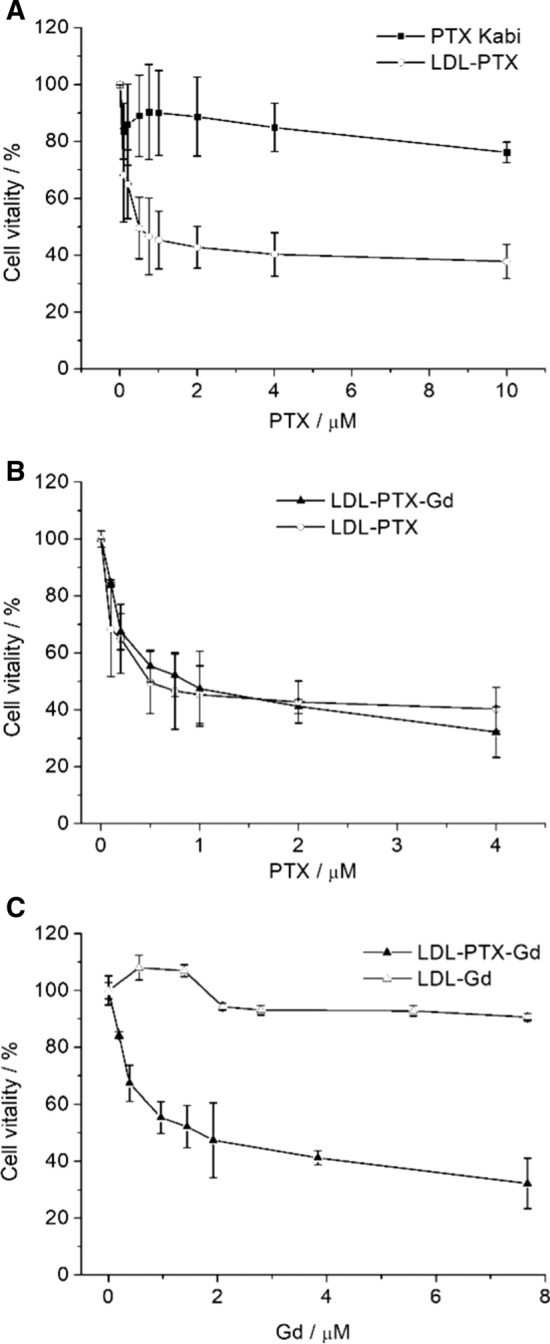
Percentage of vitality (measured by MTT assay) of B16-F10 measured after 24 h of incubation at different concentrations of Paclitaxel Kabi and LDL-PTX (**A**), LDL-PTX-Gd and LDL-PTX (**B**), and LDL-Gd (**C**). Graphs show the mean ± SD of % vitality evaluated on 3 independent experiments

Vitality assays were performed on B16-F10 by using the MTT assay. The MTT assay is a colorimetric assay for measuring cell metabolic activity. Cells were incubated for 24 h in the presence of LDL-PTX, LDL-PTX-Gd, LDL-Gd and a commercially available Paclitaxel formulation administered to human patients (Paclitaxel Kabi) at increasing concentrations (Fig. 5). The range of concentrations of PTX corresponds to plasma levels of the drug achievable in human treatments [13]. Figure 5A shows that in B16-F10 melanoma cells the cytotoxic effect is significantly higher when PTX is administered to cells loaded into LDL with respect to PTX-kabi formulation. The addition of Gd- AAZTA-C17 to the PTX-LDL adduct did not change the cytotoxic effect of the delivered drug (Fig. 5B). As expected LDL-Gd that do not contain PTX did not show any toxicity (Fig. 5C).

### Cellular uptake of LDL-Gd and LDL-PTX-Gd in B16-F10 cells

**Fig. 6:**
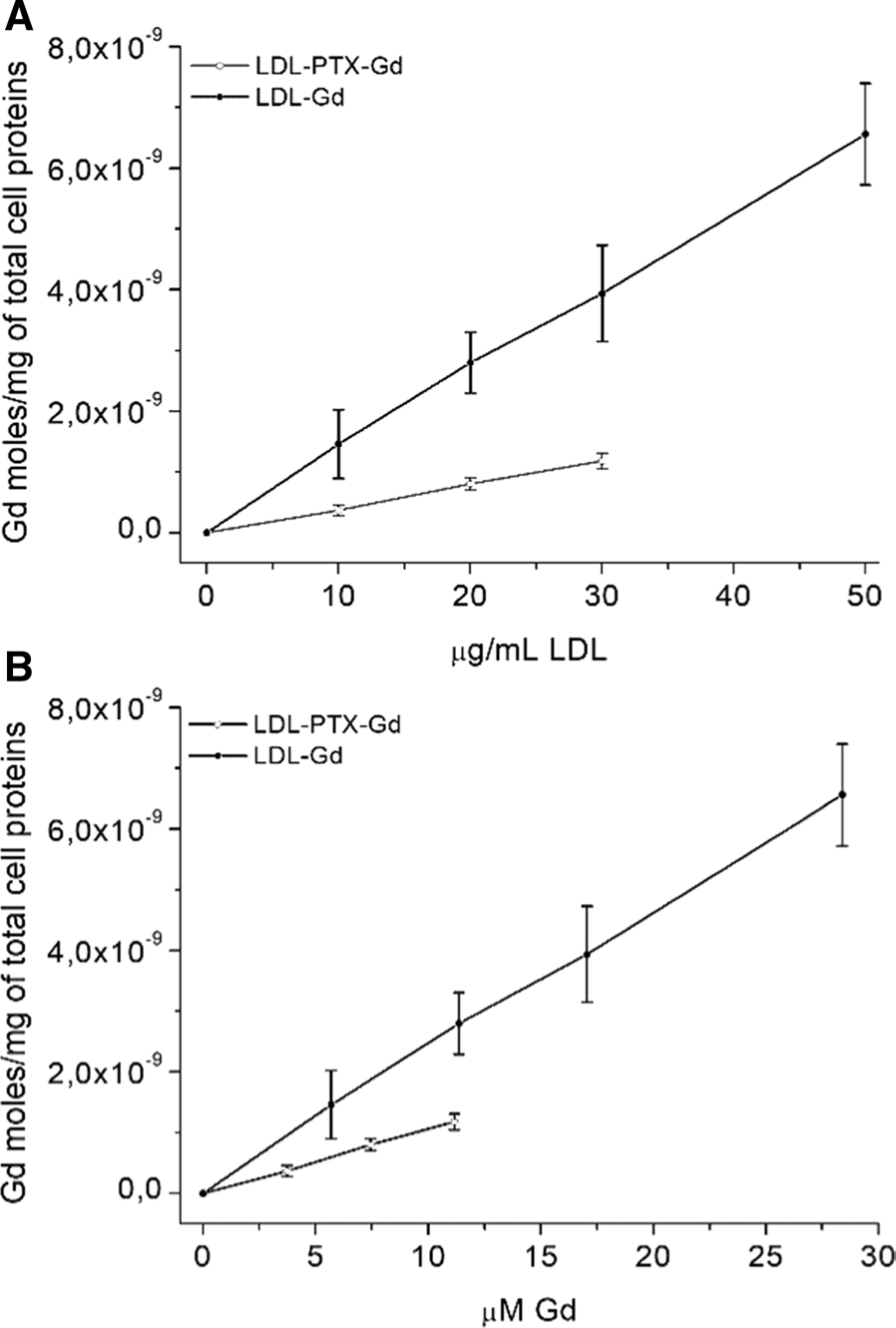
Uptake of Gd in B16-F10 cells incubated in the presence of increasing concentration of LDL-Gd (black circle) and LDL-PTX-Gd (white circle) for 24 h at 37 °C. Gd moles/mg of total proteins are reported **A** vs LDL concentration (μg/ml) or **B** vs Gd concentration (μM); Error bars indicate the SD evaluated on three independent experiments

As established above, B16-F10 express a relatively high concentration of LDL receptors on their cytoplasmatic membrane, able to internalize LDL. Cells were incubated in the presence of LDL-Gd and LDL-PTX-Gd at different concentration for 24 h at 37 °C and 5% CO_2_. Cells were then washed with cold PBS, collected and the amount of internalized Gd was measured by ICP-MS. The amount of Gd is taken as a reporter of the extent of cell internalized LDL nanoparticles. In order to compare different experiments, the amount of internalized Gd was normalized to the total protein cell content. The values of internalized Gd were higher for B16-F10 incubated in the presence of LDL-Gd than incubation of LDL-PTX-Gd (Fig. 6) accordingly to the higher Gd content in the adduct that do not contain PTX and to the toxic effect of the internalized drug that can alter cellular metabolism. The higher uptake of LDL-Gd was observed both when the Gd internalized is plotted as a function of LDL (Fig. 6A) concentration and of Gd concentration (Fig. 6B).

### Magnetic resonance imaging (MRI)

**Fig. 7:**
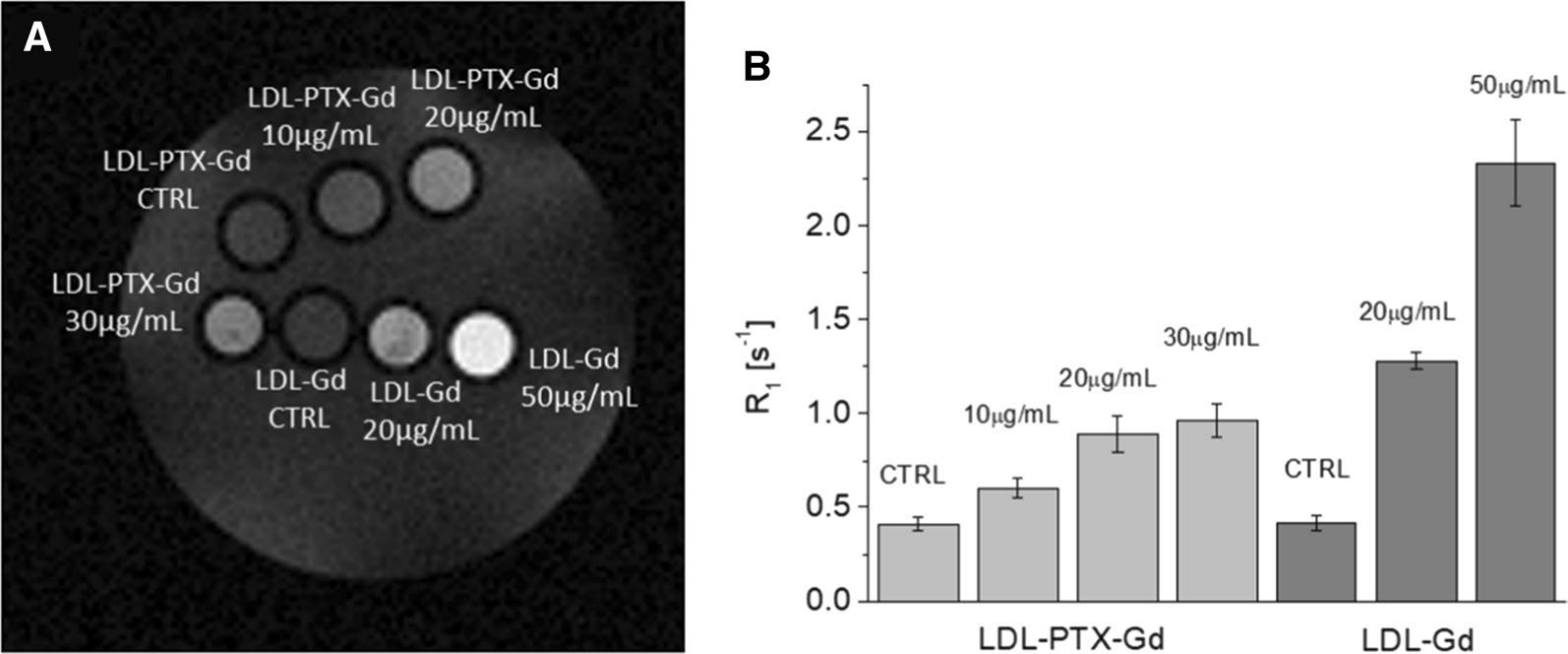
**A** T_1_-weighted spin-echo MR image of an agar phantom with glass capillaries containing untreated B16 control cells and cells incubated with 10, 20 and 30 µg/ml of LDL-PTX-Gd and with 20 and 50 µg/ml of LDL-Gd for 24 h at 37 °C; all the concentrations are referred to the µg/ml of protein (LDL) incubated **B** the corresponding relaxation rates (R_1_) measured on cell pellets for unlabeled control B16 cells (CTRL) and cells incubated with 10, 20 and 30 µg/ml LDL-PTX-Gd and 20 and 50 µg/ml of LDL-Gd; R_1_ values are reported in Additional file 1: Table S1. Error bars indicates ± SD

In order to assess whether the amount of LDL-Gd and LDL-PTX-Gd taken up by B16-F10 cells can generate a well detectable MRI contrast, T_1_- weighted images were acquired at 7 T on glass capillaries filled with B16-F10 cell pellets. Figure 7 shows the well detectable signal intensity (SI) enhancement of cells incubated in the presence of LDL-Gd or LDL-PTX-Gd at different concentrations in comparison with untreated control cells.

Being known the PTX/Gd ratio present in the LDL adduct, the amount of PTX taken-up by B16-F10 can be easily indirectly obtained by measuring the relaxation rates of cell pellets. In fact, the relaxation rates (R_1_) measured on cells pellets (Fig. 7B) are directly proportional to the product of the Gd concentration and its millimolar relaxivity at 7 T (r_1p(in cell)_ = 6.14 mM^−1^ s^−1^) (Eq. 4). Using the measurement of R_1_ enhancement of cell pellets it was possible to calculate the amount of Paclitaxel theoretically taken-up by B16-F10 (Eq. 5). The results are reported in Table 2.

4 $${\text{R}}_{{{\text{1post}}}} = \, \left[ {{\text{Gd}}} \right]{\text{mM x r}}_{{{\text{1p}}({\text{in cell}})}} + {\text{ R}}_{{{\text{1d}}({\text{cell}})}}$$

**Table 2:** Calculation of internalized PTX in B16-F10 cell line

[LDL] incubated with B16-F10	nmol PTX incubated	nmol PTX internalized	nmol/ml PTX internalized	% internalized
10 µg/ml	3.88	0.078	16.56	2.0
20 µg/ml	7.76	0.24	40.7	3.1
30 µg/ml	11.6	0.30	47.8	2.6

where R_1d(cell)_ and R_1post_ are the relaxation rates measured on cell pellets before and after the incubation with LDL-PTX-Gd.

Being the [Gd]/[PTX] ratio = 1.9 (Table 1) and R_1d(cell)_ the relaxation rate measured on untreated control cells.

5 $$\left[ {PTX} \right] = \frac{{R_{{1\left( {post Gd} \right)}} - R_{{1d\left( {cell} \right)}} }}{1.9*r1p}$$

### In vivo diagnostic and therapeutic study

**Fig. 8:**
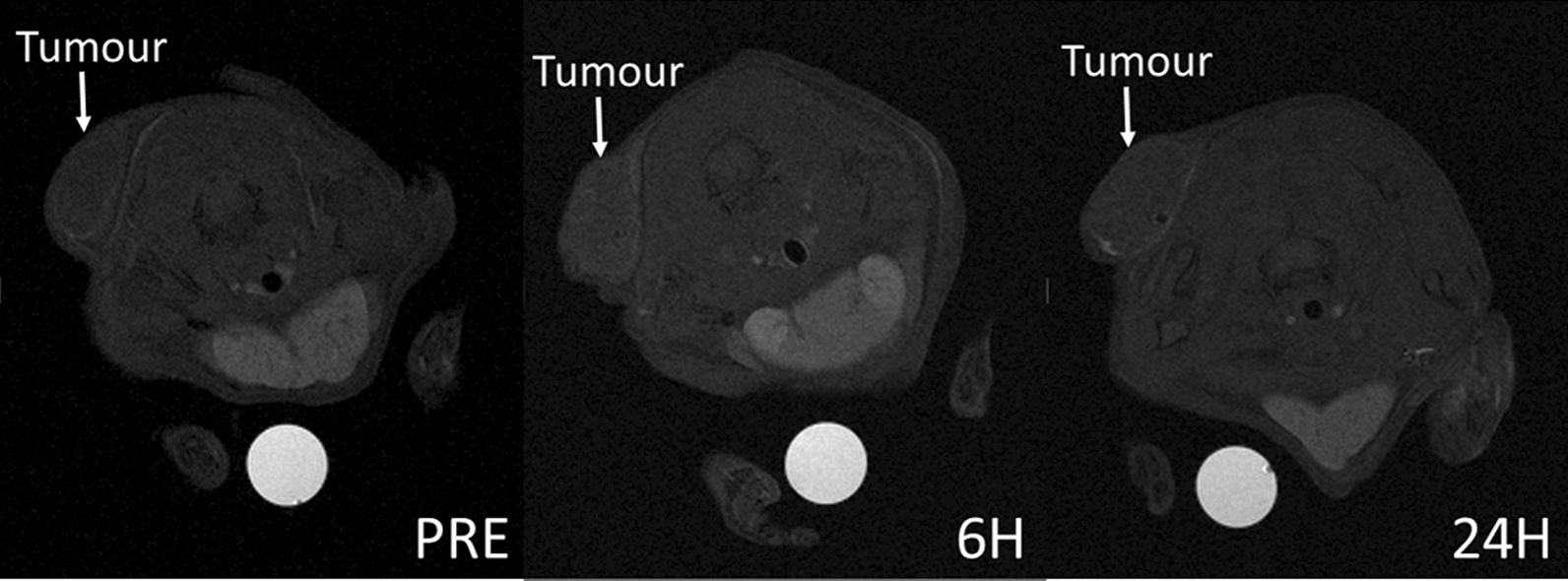
T_1_-weighted spin-echo MR images (7 T) acquired before (PRE), 6 and 24 h after contrast administration

The in vivo study was carried out on a syngeneic mouse model obtained by subcutaneous implantation, of B16-F10 cells. The tumour bearing mice were prepared by injecting ca. 0.5 million B16-F10 cells subcutaneously at the bottom of the neck of C57BL/6J mice (n = 6). After 7 days, the B16-F10 tumours reached a volume of approximately 42 ± 26 mm^3^. In order to evaluate the biodistribution of PTX, and in particular the amount of drug that LDL can deliver to the tumours, mice (n = 3) intravenously received a bolus of LDL-PTX-Gd 35 μmol/kg and 15.7 mg/Kg as expressed in term of Gd and PTX, respectively. T_1_-weighted multi slice spin-echo MR images were acquired before, 6 and 24 h after contrast administration (Fig. 8).

As expected, high % SI enhancement (Table 3) is observed in the tumour region and in the liver due to the high LDLRs expression on both tumour cells and normal hepatocytes. The amount of PTX present in muscle, liver and in the melanoma tissue (Table 4) was calculated from the SI enhancement using the equations described in material and methods. Interestingly, the amount of PTX found in tumours at 6 h is similar to the PTX internalized by B16-F10 cells in vitro after 10 μg/ml incubation of LDL (LDL-PTX-Gd).

**Table 3:** SI % enhancement measured on different organs 6 and 24 h after LDL-PTX-Gd administration

Tissue	6 h (SI % enhancement) ± SD	24 h (SI % enhancement) ± SD
Tumour	23.3 ± 1.0	2.9 ± 11.5
Muscle	12.8 ± 5.3	1.2 ± 4.7
Liver	151.2 ± 36.7	27.9 ± 24.2
Spleen	39.7 ± 13.0	15.1 ± 7.6
Kidneys	15.3 ± 9.4	-3.6 ± 1.9

**Table 4:** Amount of in vivo PTX internalized after LDL-PTX-Gd administration

Tissue	6 h PTX nmol/g ± SD	24 h PTX nmol/g ± SD
Tumour	15.0 ± 0.70	1.85 ± 7.4
Muscle	7.5 ± 2.5	0.04 ± 2.5
Liver	558 ± 38	65 ± 38

On this basis, due to the high uptake of PTX in the liver and in order to avoid a liver acute toxicity, we decided to divide the total dose in 5 different treatments, once a day for 5 consecutive days as reported in many recently published protocols [24, 25]. To perform the treatment study, a total number of 20 mice were injected with 0.5 million B16-F10 cells as described above. The mice were then rando ml y divided into three groups. The first group (n = 5) received the LDL-PTX, the second group (n = 7) was treated with PTX-Kabi, and the third (n = 8), as control, was treated with equal volume of PBS. Each mouse received in the tail vein a dose of LDL-PTX and PTX-Kabi corresponding to 4 mg/kg PTX once per day for 5 consecutive days. Mice were monitored three times weekly for body weight and for evidence of tumour or PTX treatment–associated morbidity till the end of the experiment (Additional file 1: Figure S2).

**Fig. 9:**
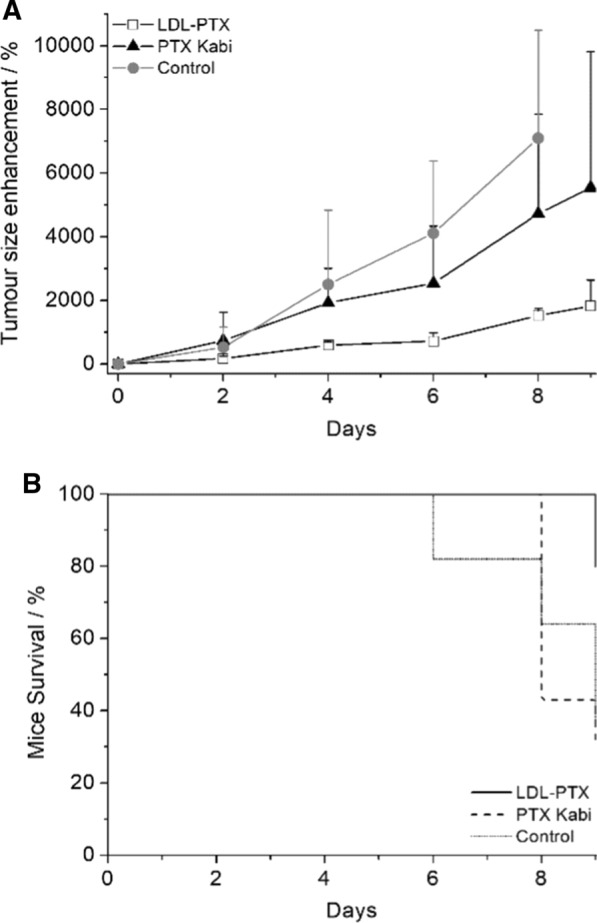
**A** Tumour-growth evaluation performed after LDL-PTX or PTX Kabi treatment. The graph shows the % of tumour size enhancement measured by MRI on untreated control mice, LDL-PTX or PTX Kabi treated mice. Error bars indicate the SD. At day 2, 4, and 6, P-values were 0.19, 0.022, 0.05, respectively. **B** Mice survival curve performed on untreated control mice, LDL-PTX or PTX Kabi treated mice

**Fig. 10:**
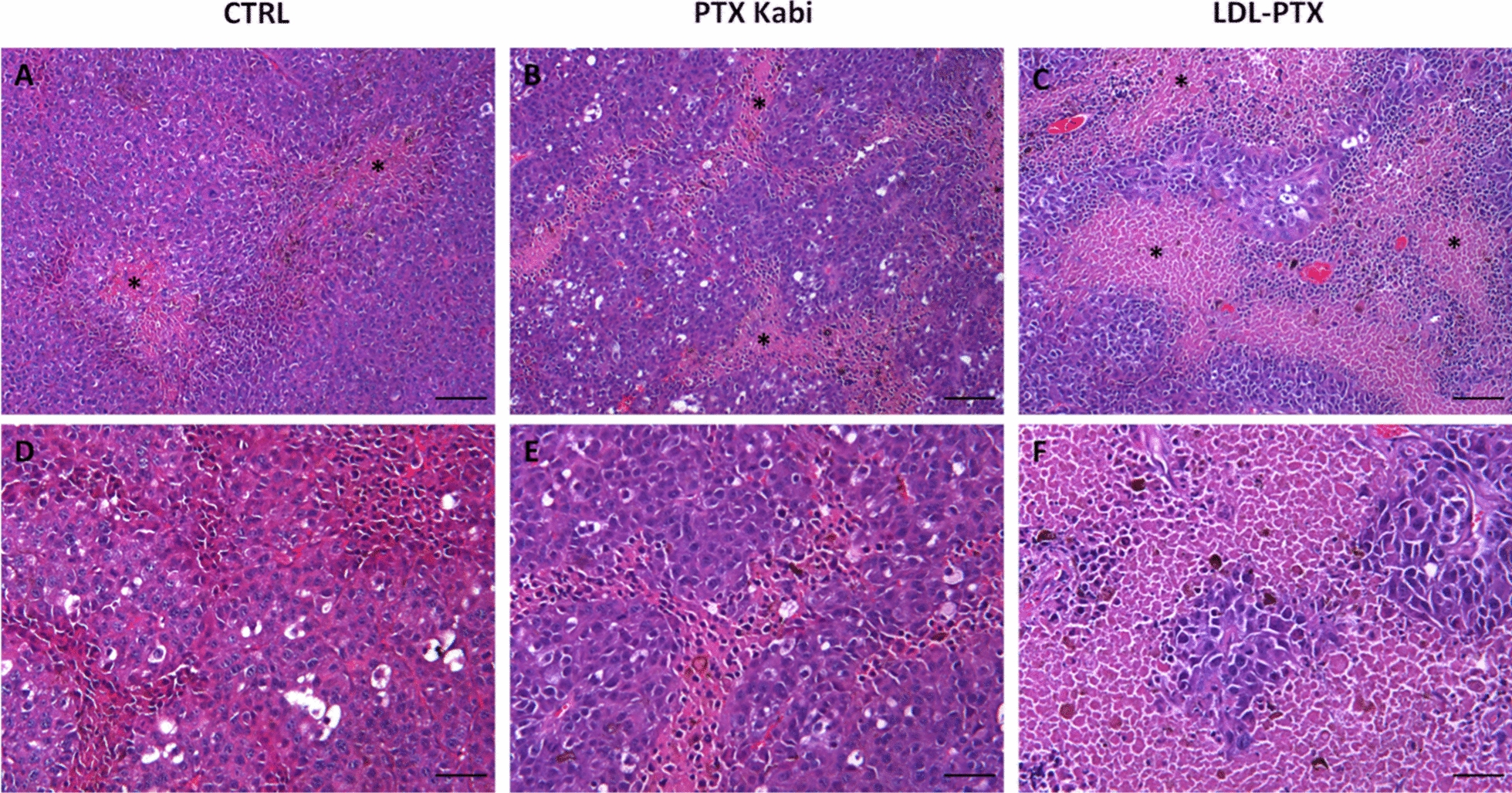
Representative H&E images of excised B16-F10 untreated control tumours (**A**, **D**), or treated with PTX Kabi (**B**, **E**) or LDL-PTX (**C**, **F**) nanoparticles. **A**–**C** Asterisks indicate necrotic tissue. **A**, **B**, **C** Scale Bars = 100 μm, Magnification ×100; **D**, **E**, **F** Scale Bars = 50 μm, Magnification ×200

Figure 9A and Additional file 1: Figure S3 show that the tumour size of mice treated with LDL-PTX monitored by MRI (at 1 T) was significantly lower than that of mice treated with PTX-Kabi or PBS. Interestingly, the high variability observed on the PTX-Kabi group was significantly reduced using the personalized delivering with LDL-PTX. At the end point, mice were euthanized and tumours were excised and undergone to ex vivo histological analysis. ImageJ analysis of ROIs manually drawn on the whole excised tumour image H&E stained, showed that the percentage of necrotic area with respect to the total tumour area was ca. 15, 30, 40% respectively for untreated control tumours, PTX Kabi and LDL-PTX. Figure 10 displays a representative magnification of the necrotic areas in the differently treated tumours.

## Discussion

The general aim of this work is to establish a new theranostic (therapeutic and diagnostic) approach based on the use of LDL for the simultaneous delivery of both antitumour drug and imaging agents. Herein developed system was tested on melanoma cells (B16-F10) that is a well known, extremely aggressive and metastatic tumour [26]. The development of procedures that lead to the accumulation of drugs at the targeting site is an important task for personalized medicine applications [27]. In this paper LDLs were exploited to deliver simultaneously different compounds inside the pathological cells, for developing an efficient antitumour theranostic nano-system. The crucial objective for MRI, as it is less sensitive with respect to other imaging modalities, is to use highly sensitive contrast agents compatible with the resulting thermodynamic stability [28, 29]. Furthermore, the pharmacological activity of a drug can be largely improved by an intracellular drug delivery. Among the different classes of Gd-based MRI contrast agents, Gd-AAZTA [30, 31] has been selected because of its high sensitivity due to the coordination of two water molecules in fast exchange with the bulk. The Gd-complex is functionalized with an aliphatic carbon chain to pursue the binding to LDL in order to allow a selective delivery to tumour cells overexpressing LDLRs. The relaxivity observed after binding with LDL, due to the increase of the tumbling time of the macromolecular adduct, is high (ca 30 mM^−1^ s^−1^ at 20 MHz) and is able to generate a good contrast enhancement when internalized in tumour cells overexpressing LDL receptors as murine melanoma (B16-F10) cell line. Paclitaxel, the antitumour drug selected for the treatment is highly hydrophobic with an extremely low solubility in water. For these reasons we propose LDL, the proteins devoted to lipids transport, as a selective carrier of natural origin. The herein developed LDL-PTX-Gd has the aim of delivering a precise therapy that can be followed in real time by in vivo imaging. In fact, Fig. 9 shows that the antiproliferative action of PTX in vivo is more effective and more reproducible in the mouse group as a consequence of its specific intracellular delivery through LDLRs. The obtained results appear very promising as they may potentially provide patients affected by melanoma with an improved therapeutic option based on the LDL driven delivery. In fact, the results reported in this article demonstrates an improved cytotoxic effect in B16-F10 tumours cells compared to commercial Paclitaxel Kabi administered alone (Fig. 5A) both in in vitro and in vivo. This interesting observation demonstrated the specific accumulation of PTX loaded LDL driven by LDL receptors. Furthermore, Fig. 5B shows that the toxicity of PTX in form of LDL-PTX but also within the LDL-PTX-Gd is the same, demonstrating the absence of toxicity due to Gd by itself. This is an important point, due to the recent alerts caused by the observation of tiny amounts of Gd may retained in the tissues of patients injected with Gd based contrast agents [32, 33]. These observations bring a growing concern about the fate of the Gd ion after the injection in the patient, despite serious consequences have been observed only for patients with impaired renal function, Furthermore, herein it has been demonstrated that by measuring the relaxation rate or simply the signal intensity enhancement of the corresponding MRI image, it is possible to extrapolate the PTX distribution in real time in a noninvasive and repeatable protocol. This potentially allows to the oncologist to have information needed to obtain an early detection of therapeutic outcome. The cytotoxicity of the LDL-PTX adduct was tested in vivo together with the evaluation of drug distribution. The specific delivery of the PTX loaded LDL in B16-F10 tumour bearing mice improved the specific delivery of PTX with respect to PTX Kabi thus markedly reducing the tumour growth. The cytotoxic effect was confirmed by histological studies on excised tumour tissues.

## Conclusions

In this study the adduct formed by loading of LDL with Gd-AAZTA-C17 and Paclitaxel was prepared and characterized for the first time to our knowledge. This therapeutic and at the same time diagnostic approach appears to have a great potential in providing highly specialized, more potent and safer tools to treat cancer. The use of endogenous biomolecules is a successful strategy for achieving this goal. Indeed, these molecules are ideal for the development of drug- delivery platforms, thanks to their biocompatibility and biodegradability. Thanks to the high uptake of LDL-PTX-Gd in in vitro and in vivo in melanoma cells we observed a significant therapeutic improvement with the advantage of monitoring the co- loaded Gd complex by MRI.

## Supplementary Information

**Additional file 1: Figure S1.** Size distribution by number, performed by DLS. Size of Native LDL (A), Size of LDL in LDL-Gd (B), in LDL-PTX (C) and in LDL-PTX-Gd (D). **Figure S2.** % Mice weight enhancement performed after LDL-PTX or PTX Kabi treatment or on untreated control mice. Error bars indicate the SD. **Figure S3.** Representative T_**2**_ weighted images (1 T) control, PTX Kabi and LDL-PTX treated mice, monitored by MRI (7 T) on day 0 and 4. **Table S1.** R_1_ values measured on cell pellets of Fig. 7.

## Data Availability

Not applicable.
