# Supplementary material for: LDL mediated delivery of Paclitaxel and MRI imaging probes for personalized medicine applications
Source: J Nanobiotechnology. 2021 Jul 13;19:208. doi: 10.1186/s12951-021-00955-9 (PMC8276427; doi:10.1186/s12951-021-00955-9)
Supplement: Supplementary file 1 — Additional file 1: Figure S1. Size distribution by number, performed by DLS. Size of Native LDL (A), Size of LDL in LDL-Gd (B), in LDL-PTX (C) and in LDL-PTX-Gd (D). Figure S2. % Mice weight enhancement performed after LDL-PTX or PTX Kabi treatment or on untreated control mice. Error bars indicate the SD. Figure S3. Representative T2 weighted images (1 T) control, PTX Kabi and LDL-PTX treated mice, monitored by MRI (7 T) on day 0 and 4. Table S1. R1 values measured on cell pellets of Fig. 7. [file 12951_2021_955_MOESM1_ESM.docx]

**Additional Material**

**LDL mediated delivery of Paclitaxel and MRI imaging probes for personalized medicine applications**

Sahar Rakhshan^a#^, Diego Alberti^a#^, Rachele Stefania^a^, Valeria Bitonto^a^, Simonetta Geninatti Crich^a*^

*^a^Department of Molecular Biotechnology and Health Sciences, University of Torino, via Nizza 52, Torino, Italy.*


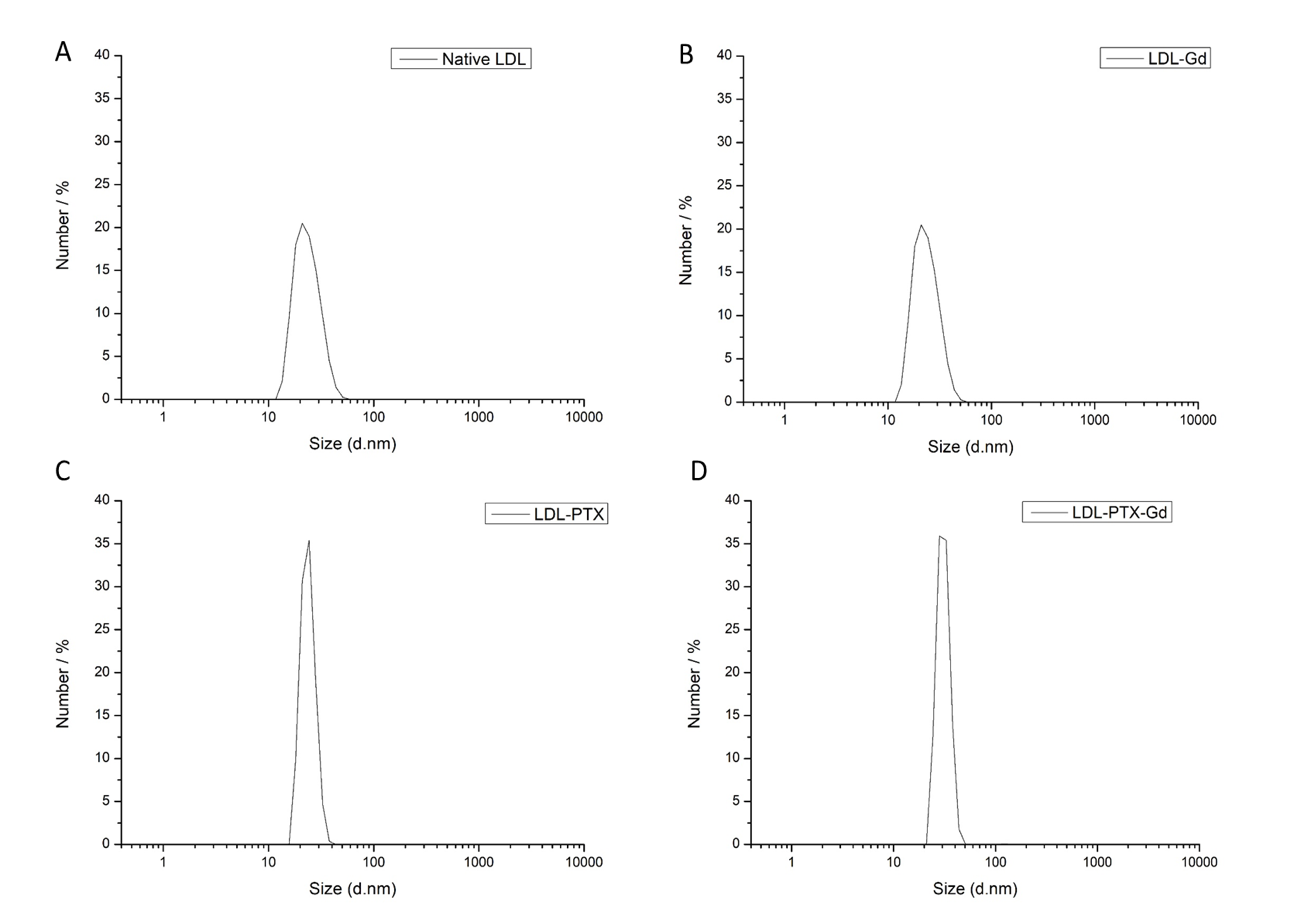


**Figure S1**. Size distribution by number, performed by DLS. Size of Native LDL (A), Size of LDL in LDL-Gd (B), in LDL-PTX (C) and in LDL-PTX-Gd (D).





**Figure S2**. % Mice weight enhancement performed after LDL-PTX or PTX Kabi treatment or on untreated control mice. Error bars indicate the SD.

**
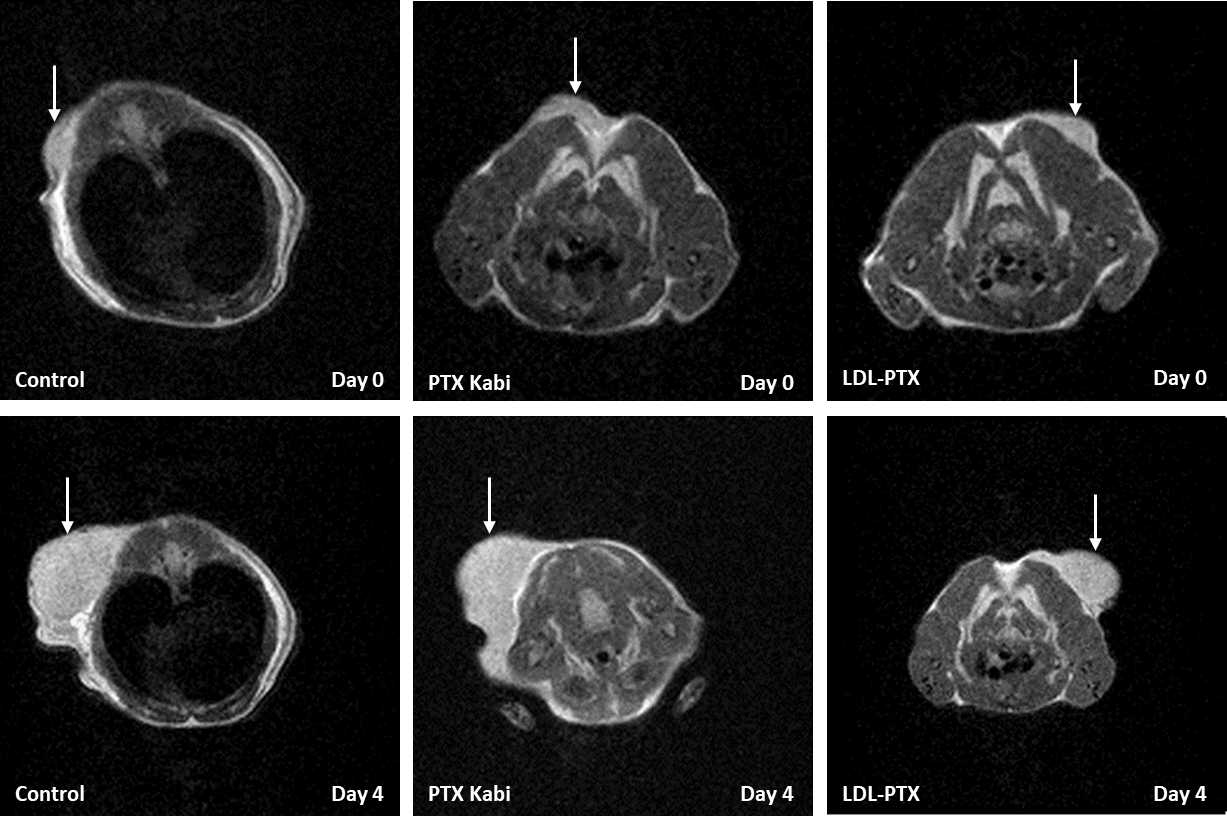
**

**Figure S3**. Representative T**_2_** weighted images (1 T) control, PTX Kabi and LDL-PTX treated mice, monitored by MRI (7 T) on day 0 and 4.

| **Experiment** | **Cell samples** | **R_1_ [s^-1^] ± SD** |
| --- | --- | --- |
| LDL-PTX-Gd | control | 0.410±0.037 |
|  | 10 µg/mL LDL | 0.603±0.053 |
|  | 20 µg/mL LDL | 0.891±0.097 |
|  | 30 µg/mL LDL | 0.965±0.087 |
| LDL-Gd | control | 0.418±0.040 |
|  | 20 µg/mL LDL | 1.280±0.044 |
|  | 50 µg/mL LDL | 2.336±0.232 |

**Table S1**. R_1_ values measured on cell pellets of Figure 7.
